# Promoting hand hygiene in the Asia Pacific region

**DOI:** 10.1186/1753-6561-5-S6-O69

**Published:** 2011-06-29

**Authors:** ML Ling, WH Seto, TY Ching, G Harrington, ML Grayson, D Pittet

**Affiliations:** 1 Infection Control, Singapore General Hospital, Singapore, Singapore; 2 Infection Control, Hospital Authority Head Office,Hong Kong, China; 3 Infection Control, WHO Collaborating Center, Hong Kong, Hong Kong, China; 4 Infection Control Consultancy, Austin Health, Melbourne, Australia; 5 Infectious Diseases, Austin Health, Melbourne, Australia; 6 Infection Control Program, University of Geneva Hospitals, Geneva, Switzerland

## Introduction / objectives

Since 2003, countries in the Asia Pacific region understood the importance of a good infection control program following the SARS and Pandemic Flu experiences. However, with limited resources, the challenge remains how one can creatively promote good hand hygiene compliance to prevent the transmission of pathogens along with meeting other important core components of an infection prevention program.

## Methods

In 2009 the Asia Pacific Society of Infection Control collaborating with Hôpitaux Universitaires de Genève and Aesculap Academy invited countries in the Asia Pacific region to participate in the Asia Pacific Hand Hygiene Excellence Award. This is a platform to recognize, honor, and celebrate those hospitals and healthcare-worker groups who have made use of their enthusiasm and creativity to improve patient safety through the successful implementation of the WHO multimodal strategy in their hospitals.

## Results

For 2010 there are 8 finalists - 3 from Indonesia, 2 from Singapore and 1 each from Australia, Vietnam and India. Innovative ideas were noted in the applications received. In site visits made at potential winner institutions, strong leadership was evident in giving rise to their vibrant programs. These use the Guide to Implementation of the WHO Multimodal Hand Hygiene Improvement Strategy to help them prepare effective Action Plans. Awards will be given to institutions who achieved high scores utilising the WHO Assessment Framework Tool.

## Conclusion

The requirements for the award have encouraged many institutions to enhance their hand hygiene program accordingly and we anticipate a larger participation for the 2011 award.

## Disclosure of interest

None declared.

